# A systematic review of condition-specific preference-based measures used in young people and their valuation methods

**DOI:** 10.1186/s41687-024-00826-5

**Published:** 2024-12-19

**Authors:** William King, Lauren Hockley, Tomos Robinson, Angela Bate, Laura Ternent

**Affiliations:** 1 Health Economics Group, Newcastle University Population Health Sciences Institute, Newcastle upon Tyne, UK; 2 Newcastle upon Tyne Hospitals NHS Foundation Trust, NHS, Newcastle upon Tyne, UK; 3 Northumbria University, Newcastle upon Tyne, UK

**Keywords:** Systematic review, Health-related quality of life, Preference elicitation, Economic evaluation

## Abstract

**Background:**

Condition-specific health-related quality-of-life (HRQoL) instruments can be more responsive and sensitive to specific conditions and diseases than generic HRQoL instruments. This systematic review aims to identify the condition-specific preference-based instruments that have been used with young people and identify how preference values have been obtained for these instruments. This review will inform future researchers about the methods used to elicit utilities for condition-specific HRQoL instruments.

**Methods:**

A comprehensive search strategy was used to identify condition-specific HRQoL instruments used in young people and the methods used to value these instruments. Published medical and health economic databases were searched from January 1990–March 2022. Articles were deemed eligible for inclusion if a condition-specific preference-based instrument was used in young people (age < 18). Screening, data extraction and quality assessment were conducted independently by at least two reviewers.

**Results:**

After deduplication, a total of 4273 articles were eligible for title and abstract screening. Of these, 98 articles were eligible for full-text screening. After full-text screening, 18 articles were included in the review. Valuation studies were the most prevalent study design in the review (44%), followed by mapping studies (38%) and then other designs (18%). Among the valuation studies, the choice of HRQoL instrument, preference elicitation method, anchoring method and perspective varied considerably.

**Conclusion:**

To our knowledge, this review is the first to explore what condition-specific HRQoL instruments have been used in young people. Findings from this review could inform researchers in their choice of methods for measuring and valuing HRQoL. This review illustrates that to date there does not appear to be clear consensus of how to measure and value HRQoL in young people when using condition-specific instruments. The lack of consensus could be influenced by challenges identified in prior research such as limited guidance, ethical issues, and uncertain normative decisions regarding the choice of preference elicitation method. Ordinal methods such as discrete choice experiment and best-worst scaling appear to be preferable for use in this population.

**Supplementary information:**

The online version contains supplementary material available at 10.1186/s41687-024-00826-5.

## Introduction

The aim of this systematic review is to identify condition-specific preference-based instruments used to measure and value health-related quality of life (HRQoL) in young people (age < 18). As evidenced in the Rowen et al. [1] review, considerable variation in methodological approaches in the measurement of young people’s HRQoL exists in terms of both the variety of instruments and different preference elicitation methods. This variation in methodological approach could, in part, be explained by the lack of clear guidance from international agencies and national organisations on how to generate quality adjusted life-years (QALYs) for young people which can be used in economic evaluations.

The systematic review conducted by Rowen et al. [1] identified a number of generic preference-based patient reported outcome measures (PROMs) that have been specifically developed for use in young people. However, the use of condition-specific PROMs and their valuation methods has been less explored in the evidence base. Condition-specific PROMs have a key advantage over generic PROMs in that they can be considered more responsive and sensitive to specific conditions and diseases than generic PROMs [2]. This is especially evident in areas such as optometry [3] and asthma [2] where generic PROMs struggle to identify meaningful differences in the HRQoL of respondents. One area in which condition-specific instruments could be advantageous is in the area of childhood obesity. Oluboyede and Robinson [4] assessed the sensitivity of a generic instrument, the Child Health Utility 9-Dimension (CHU-9D), compared to a condition-specific instrument, the Weight-specific Adolescent Instrument for Economic evaluation (WAItE), and found that the WAItE was more sensitive at measuring change in HRQoL and is better suited to measuring HRQoL in this particular disease area. In this study, the WAItE exhibited lower ceiling effects than the CHU-9D, with the authors concluding that it is more likely to pick up meaningful changes in HRQOL, particularly at the higher end of the distribution [4]. Additionally, Crump et al. [5] found that condition-specific instruments exhibit higher construct validity than generic instruments. Therefore, in some contexts, there are key advantages in terms of sensitivity for using condition-specific instruments. This potential sensitivity gain has led to the development of a number of condition-specific preference-based instruments [6–10] to better measure young people’s HRQoL. While a number of these instruments have now been developed, a full review of condition-specific preference-based instruments is yet to be completed and this study aims to address this gap in the evidence base.

This review will also present and evaluate preference elicitation methods used to value responses to these condition-specific HRQoL instruments. For instruments to be considered preference-based, they should be able to generate utility values based on the responses to a descriptive system. Methods to convert these results from the descriptive system into utilities vary widely, and accepted practice has varied over time. Visual analogue scale, standard gamble, time trade-off, discrete choice experiment (DCE) and best-worst scaling (BWS) are all examples of preference elicitation task that have been used to conduct valuation studies which seek to ‘value’ results from a descriptive system. Methods used in these preference elicitation tasks vary widely between studies and a number of methodological questions remain unanswered in terms of whose preferences are relevant, what perspective respondents should consider and how should these preferences be anchored onto the 0–1 QALY scale (where a score of zero corresponds to being dead and a score of 1 refers to full health). Another approach which does not utilise preference elicitation methods is to conduct a mapping or ‘cross-walking’ study. A mapping study uses econometric methods to generate an algorithm that can be used to predict health state utility values from an existing preference-based instrument [11]. A mapping exercise is considered to be a second-best exercise compared to either the direct use of a generic preference-based instrument or a valuation of the condition-specific instrument [12]. Mapping studies will be included in this review alongside valuation studies based on preference elicitation methods.

This systematic review will identify and present which condition-specific preference-based instruments have been used in young people and which preference elicitation methods have been used to elicit preference values for these instruments.

## Methods

This systematic review was reported in accordance with the Preferred Reporting Items for Systematic Review (PRISMA) guidelines [13]. A prespecified protocol [14] was published on PROSPERO database of systematic reviews.

### Search strategy

A comprehensive search strategy was used. The search strategy was informed by the Cochrane Collaboration guidelines on search strategies for economic evaluations [15], previous reviews of preference elicitation methods [1, 5] and using the York Health Economics Consortium (YHEC) health state utility values filter [16]. The search strategy used is presented in Appendix Table 4.

Databases of published, peer-reviewed medical or health economic research were searched: Ovid Medline, School of Health and Related Research Health Utilities Database (ScHARRHUD), National Health Service Economic Evaluation Database (NHS EED), Cost-Effectiveness Analysis (CEA) registry and the Health Technology Assessment (HTA) Database. The database search was restricted to papers published since 1st January 1990 until the 1st March 2022. More recent papers which were identified through citation chasing were also included. Searches were restricted to include only studies published in the English language.

### Inclusion criteria

Articles were deemed eligible for inclusion if a condition-specific quality of life instrument that was designed for use in young people (age < 18) was used. Included studies were required to use instrument responses from young people to estimate QALYs. Adult responses to preference elicitation (valuation) studies were also included. Preference elicitation studies (valuation studies), mapping studies and economic evaluations were included. Articles were excluded if young people’s HRQoL was estimated using a generic HRQoL instrument such as the EQ-5D, HUI, CHU9D or SF-36. For mapping studies, a condition-specific instrument must have been used to measure HRQoL. While mapping condition-specific instruments onto generic instruments may not be the most sensitive approach to eliciting utilities, we have included these studies in this review for completeness. Studies which mapped body mass index to HRQoL were not included. Additionally, systematic reviews and meta-analyses were excluded.

### Study screening and selection

Comprehensive screening of the database searches was carried out independently by five reviewers (WK, LH, TR, LT, AB). Rayyan software was used to conduct all study screening [17]. One author (WK) reviewed all title and abstracts, with a second reviewer (LH) independently reviewing a 10% random sample. All full texts were screened by one author (WK) and independently screened by a second reviewer. Second reviewers (LH, TR, LT, AB) each screened 25% of full-texts. Conflicts between reviewers were resolved through moderation with the main reviewer (WK). Citation chasing was conducted using reference lists from included papers and upcoming studies referred to in included papers.

### Data extraction

A data extraction form was developed in Microsoft Excel [18] to capture relevant methodological information. Data extraction was conducted by one author (WK) and a proportion (10%) was checked by a second reviewer (LH). Table 1 presents the extracted information.

**Table 1:** Data extracted from papers

Data extracted			
Authors	Study type	HRQoL instrument	Self/proxy reported
Title	Condition area	Valuation method	Anchoring method
Data extracted	Sample size	Valuation respondent	Source of utility values
Country	Population age	Perspective adopted	Other details

### Quality assessment

Due to heterogeneity in study design among included studies, it was necessary to use two different quality assessment tools to accommodate for these different study designs. Studies which met the inclusion criteria were quality assessed using one of two specified tools depending on their study design: REporting invenToRy chIld hEalth ValuEs (RETRIEVE) [19] and MApping onto Preference-based measures reporting Standards (MAPS) [11]. The RETRIEVE tool was used to quality assess papers that estimated health state utility values via a valuation study while the MAPS tool was used to quality assess papers that estimated health state utility values using a mapping algorithm.

## Results

**Fig. 1:**
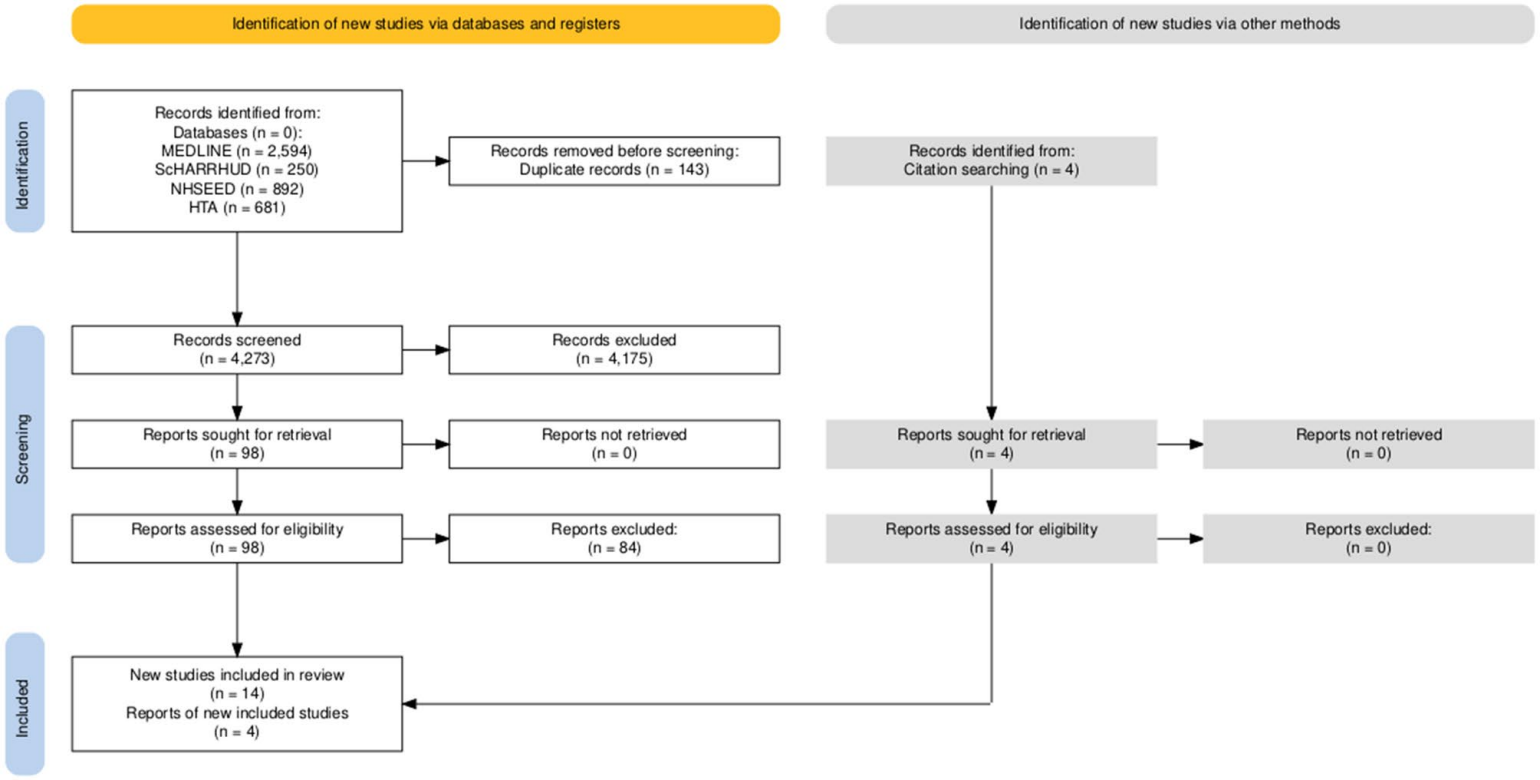
PRISMA diagram

A total of 4416 articles were identified through database searching. After removal of duplicates, there were a total of 4273 articles eligible for title and abstract screening. Of these, 4175 articles were excluded which left 98 articles eligible for full-text screening. During the full-text screening, four articles were identified through citation chasing and included in the review. After the elimination of 84 articles at the full-text screening stage, this left a total of 18 articles included in the review. The qualitative synthesis was informed by these 18 included articles. Figure 1 displays the Preferred Reporting Items for Systematic reviews and Meta-Analyses (PRISMA) diagram [13, 20] detailing the database searching and screening process. Table 2 presents summary information from included studies in the review. Included studies are presented in Appendix Table 5.

**Table 2:** Study summary information

First author (year)	Country	Study design	Condition area	Population age	HRQoL instrument	Self/proxy reported
Hettiarachchi, RM (2023)	Australia	Valuation	Oral health	*>*18	ECOHIS-4D	Proxy
Tonmukayakul, U (2020)	Australia	Mapping	Cerebral palsy	6–15 yrs	CPQoL-Child	Proxy
Sharma, R (2019)	Australia	Mapping	Behavioural screening questionnaire	10–15 yrs	SDQ	Self
Kulkarni, A (2004)	Canada	Mapping	Hydrocephalus	Mothers of 5–17 yrs	HOQ	Proxy
Reckers-Droog, V (2020)	Netherlands	Measure developmnt	Youth mental health	12–18 yrs	ASC T-ASI	Self
Rogers, H (2019)	UK	Valuation	Oral health	11–16 and *>*18	CARIES-QC–U	Self
Beusterien, K (2012)	UK	Valuation	Hunters syndrome/chronic conditons	*>*18	AHUM	Self
Robinson, T (2019)	UK	Mapping	Overweight and obesity	Adolescents	WAItE	Self
Oluboyede, Y (2013)	UK	Valuation	Obesity	*>*18	WAItE	Self
Stevens, K (2005)	UK	Valuation	Atopic dermatitis	*>*18	QoLIAD	Self
Retzler, J (2018)	UK	Valuation	Allergic rhinoconjunctivitis	8–11 yrs and *>*18 yrs	Allergic rhinoconjunctivitis and asthma health states	NS
Payakachat, N (2014)	US	Mapping	Autism spectrum disorders	4–17 yrs	Child Behaviour Checklist (CBCL)	Proxy
Wright, D (2016)	US	RCT	Mental health	13–17 yrs	Child Depression Rating Scale	Self
Chiou (2005)	US	Valuation	Antisocial behaviour	*>*18 yrs	Behavioural Prob-lems Index (BPI)	Proxy
Petrou, S (2014)	UK	RCT	Asthma	2–16 yrs	PedsQL Asthma Scales	Proxy
Wong, C (2017)	China	Mapping	Scoliosis	NS	SRS-22r	Self
Kulkarni, A (2003)	Canada	Mapping	Hydrocephalus	NS	HOQ	Self
Schawo, S (2019)	Netherlands	Valuation	Addiction	18–65 yrs	ASC T-ASI	Self

### Study design

A number of study designs were captured in this review including: valuation studies (44%), mapping studies (38%), RCTs (11%) and outcome measure development studies (6%). Among included studies, two predominant study designs were identified, namely stand-alone valuation studies and mapping studies. A stand-alone valuation study (hereinafter referred to as valuation study) generates values for young person’s HRQoL, including for disease-specific states and value sets for generic child HRQoL instruments [19]. Valuation studies make use of preference elicitation methods to elicit utility values from descriptive systems. Mapping studies, in this context, involve the development and use of an algorithm to predict health state utility values from a generic preference-based instrument [11].

### HRQoL instruments and valuation methods used

The HRQoL instruments used by authors differed across the included studies. As per the inclusion criteria, all studies used condition-specific instruments which focused on a particular disease area when estimating HRQoL. Table 2 presents the different HRQoL instruments used, the corresponding disease area and mode of administration (i.e. self or proxy reported).

Among valuation studies, a variety of preference elicitation methods were used. DCE with duration was the most commonly used preference elicitation method among the included valuation studies (5 studies). Followed by standard gamble (2 studies), time-trade off, BWS, standard DCE and visual analogue scale (used once each). Two studies [10, 21] elected to use a combination of valuation methods to elicit preferences depending on the valuation respondent. Retzler et al. [21] administered the standard gamble to adults and the visual analogue scale to children. Rogers et al. [10] used BWS tasks to elicit young peoples preferences while using DCE with duration to capture adult’s preferences. Table 3 presents the details of the valuation methods used by authors in the review.

**Table 3:** Valuation methods results

Valuation method	First author (year)	Valuation respondent	Perspective adopted	Self/proxy reported	Anchoring method	Source of utility values
Best-Worst Scaling	Rogers, H (2019)	Adolescents	Own	Self	DCE with duration	Direct elicitation
DCE	Schawo, S (2019)	Adults	Hypothetical adolescent	Self	Anchored PITS state at 0 and the best state at 1. Then applied latent coefficients to this 0–1 scale	Direct elicitation
DCE with duration	Rogers, H (2019)^a^	Adults	Own	Self	DCE with duration	Direct elicitation
	Hettiarachchi, RM (2023)	Adults	Respondents were asked to imagine a hypothetical child when choosing between the health scenarios provided	Proxy	DCE with duration	Direct elicitation
	Oluboyede, Y (2013)	Adults	Own	Self	DCE with duration	Direct elicitation
	Chiou (2005)	Adults	Own	Proxy	DCE with duration	Direct elicitation
Standard gamble	Retzler, J (2018)	Adults	Own for adults and other child for YP	NA	NA	Direct elicitation
	Stevens, K (2005)	Adults	Child perspective	Self	NA	Direct elicitation
TTO	Beusterien, K (2012)	Adults	Own	Self	NA	Direct elicitation
VAS	Retzler, J (2018)^a^	Children	Own for adults and other child for YP	NA	NA	Direct elicitation

^a^Where two valuation methods were conducted with different populations studies appear more than once [10, 21]

### Valuation respondent

The choice of valuation respondent varied among included studies. The most common choice of valuation respondent was adults (8 studies). There were however two studies which used children and/or young people to complete the preference elicitation tasks [10, 21]. These two studies also opted to use a combination of both child and adult preferences for completing the preference elicitation tools [10, 21].

### Valuation perspective

The most common choice of perspective among included studies was participants ‘own’ perspective (75%). The next most frequently chosen perspective was that of the hypothetical child or young people (50%) where the valuation respondent was adults. One study [21] changed the perspective depending upon whether an adult or young people was completing the preference elicitation task. Adults were asked to answer from their own perspective while young people were asked to answer from the perspective of a hypothetical child.

### Anchoring method

Anchoring methods were not employed among all studies as a number of the preference elicitation tasks (i.e. SG, TTO, VAS) did not require the use of anchoring methods. However, studies which elicited preferences on a latent scale, used an anchoring method to transform these preferences onto the 0–1 utility scale. The most common anchoring method used among authors was the DCE with duration. DCE with duration was used in various forms by authors in the review. While DCE with duration was used most commonly in isolation, Rogers et al. [10] elected to use a combination of profile-case best worst scaling methods and DCE with duration. This involved using BWS methods for eliciting preferences for the majority of health states and then using DCE with duration for the anchoring.

### Quality assessment

Quality assessment was conducted on valuation studies and mapping studies. The RETRIEVE tool [19] was used to quality assess 8 papers which met the criteria to be considered a valuation study. The MAPS tool [11] was used on 7 papers which were mapping study. Complete quality assessment checklists are presented in supplementary material Table S2.

## Discussion

This review has highlighted the variation in methods for researchers who wish to measure young people’s preferences using a condition-specific HRQoL instrument. Roughly half of studies included in this review estimated utilities using a mapping approach. This finding is interesting as it is broadly acknowledged that estimating utilities using a mapping algorithm is a second-best approach when compared to conducting a valuation study [22]. The abundance of mapping studies included in this review could be due to a number of factors. Firstly, resource constraints could play a role in the abundance of mapping studies. Valuation studies can require large sample sizes and consequently the costs of data collection may represent a barrier to authors who seek to elicit preferences [23, 24]. Secondly, conducting valuation studies requires additional choice modelling skills as a researcher whereas mapping studies can be comparatively straightforward. Therefore, task complexity could play a role in determining which study design to opt for. Finally, arguably the largest determinant for authors study design is likely to be the limited guidance and ongoing challenges surround eliciting preferences for children and young people [1]. As discussed in Rowen et al. [1], many methodological questions remain unanswered regarding the measurement and valuation of children and young people’s HRQoL. For instance, it is unclear whose preferences should be used for valuation of young people’s HRQoL. Another methodological question is the possibility, and appropriateness, of combining utilities elicited from adults and those elicited from adolescents. It is also unclear whether the age and description of the child impacts on preferences elicited by adults who are valuing from the child’s perspective. The combination of these unanswered methodological questions could be adding an additional layer of complexity for researchers who wish to conduct a valuation study with young people. Mapping studies, despite not being the gold standard in measuring HRQoL [23, 24], have the appeal of being able to circumvent these challenges by not having to directly address any of these questions and perhaps this is part of the reason they feature heavily in the literature.

The results from this review indicate that generally authors tended to vary their valuation method depending on the valuation respondent. Rogers et al. [10] varied their approach by using DCE with duration techniques for adults involved in the study while employing BWS methods for the adolescents involved. This variation was due to findings from prior qualitative research [25] which suggested that young people preferred BWS tasks and demonstrated a better understanding of the task compared to DCE tasks. Additionally, Retzler et al. [21] varied their approach through administering standard gamble to adults and visual analogue scale to children and young people. BWS and VAS are both preference elicitation tasks which do not confront participants with questions around the valuation of death and so circumvent potential ethical concerns about talking about death with children and young people. It is possible that these ethical concerns are playing a role in determining the choice of preference elicitation task by authors who wish to elicit preferences directly from children and young people.

Among studies which conducted preference elicitation tasks where adults conducted the task, DCE was the most frequently conducted. This could be due to theoretical advantages associated with adopting this method. For instance, when compared to a VAS, DCE may more accurately emulate how individuals make choices as it is an ordinal method rather than a cardinal method [26]. Ordinal methods, such as DCEs, are theoretically grounded in random utility theory which outlines how individuals make decisions under uncertainty [26]. Additionally, growing evidence [26–28] suggests that ordinal methods are comparatively easier for young people to complete and thus more appropriate for use in the younger population. The combination of these benefits associated with DCE could explain the popularity of these methods in this review.

The valuation studies identified by this review were predominantly populated with adult responses to preference elicitation tasks. This is unsurprising given the aforementioned challenges associated with eliciting children and young people’s preferences. However, emerging qualitative research on the UK public’s views on valuing HRQoL in children is suggests that children should be involved in valuation in some form, yet this should differ depending on age or maturity [29]. Therefore, in the UK at least, public opinion seems to support the involvement of children and young people in preference elicitation tasks.

While a number of studies have involved children and young people in valuation studies [26, 30, 31], the majority of these have used generic HRQoL instruments. Thus, greater effort could be made by researchers in the future to include children and young people in condition-specific HRQoL valuation studies.

Own perspective was most frequently used in this review although this varied depending on the respondent completing the preference elicitation task. Studies have illustrated that the perspective adopted by respondents does matter and can influence individual’s preferences and therefore the underlying value-sets [30, 32, 33]. Additionally, the choice of perspective has been referred to by Rowen et al. [1] as a normative decision that should be well-considered. Qualitative work by Powell et al. [29] suggests that the UK public supports the use of ‘own’ perspective rather than using a hypothetical child or hypothetical adult.

Of those studies which required the use of anchoring methods, the most common method applied was the DCE with duration. This method was used exclusively with adults in this review which could imply that it is unsuitable for use with children and young people. Applying anchoring methods in the younger population can be challenging from an ethical perspective due to many of the anchoring preference elicitation methods referring explicitly to mortality and death. These ethical issues typically arise when valuing the PITS (worst) health state which needs to be anchored to a particular preference value. Studies can and have been designed to circumnavigate around these challenges by using adult preferences to anchor young peoples responses to preference elicitation tasks on the 0–1 QALY scale. Rogers et al. [10] employed this method to develop a value-set for the CARIES-QC which is based on adolescent responses to a BWS task. Adult responses to a DCE with duration task, where the PITS health state was valued, were used to anchor the latent coefficients estimated in the BWS task with adolescents. Other studies [26] have employed similar methods to anchor young peoples responses on the 0–1 QALY scale.

This review identified only decompositional approaches to obtain utility values based on condition-specific descriptive systems. Conversely, compositional methods tend to evaluate the product attributes and levels separately, and then the total utility of a product can be computed by a simple linear aggregation rule [34]. More recently, compositional approaches have been used to obtain utility values for condition-specific HRQoL instruments. One such method which has gained momentum over recent years is the use of personal utility functions to enable the estimation of value sets. Traditionally this approach was conducted via in-person interviews however Schneider et al. [35] have more recently developed an online version so-called the Online Personal Utility Functions (OPUF). This approach involves three main constituent parts: criteria weighting the attributes, preference weighting the level rating and anchoring on the QALY scale. Since its development, the OPUF method of eliciting preferences has been used with both generic and condition-specific instruments [6, 35]. Bray et al. [6] have used the OPUF to develop a preference-based value set for the MobQoL-7D—a validated measure of mobility-related quality of life for use in adults. Using the OPUF with young people has not been conducted yet and ethical complications surrounding the acceptability of the anchoring task would require careful consideration. Despite this, the OPUF presents an interesting new approach to estimating value sets for condition-specific HRQoL instruments.

## Conclusion

This review has illustrated that there does not appear to be a clear consensus in terms of how to value HRQoL in young people when using condition-specific instruments. Research continues to be hampered by limited guidance, ethical complications and uncertain normative decisions surrounding the choice of preference elicitation method, whose preferences to use and whose perspective should be considered. Ordinal methods such as DCE and BWS appear to be preferable for use in this population. However, compositional approaches such as the OPUF could present an innovative way to circumvent some of these challenges.

**Supplementary information**: Results from data extraction are presented in supplementary material Table S1. Complete quality assessment checklists are presented in supplementary material Table S2.

## Electronic supplementary material

Below is the link to the electronic supplementary material.

Supplementary Material 1

Supplementary Material 2

Supplementary Material 3

Supplementary Material 4

Supplementary Material 5

Supplementary Material 6

Supplementary Material 7

Supplementary Material 8

Supplementary Material 9

Supplementary Material 10

Supplementary Material 11

Supplementary Material 12

Supplementary Material 13

Supplementary Material 14

Supplementary Material 15

Supplementary Material 16

## Data Availability

All data generated or analysed during this study are included in this published article (and its supplementary information files).

## Appendix A Search strategy

**Table 4:** MEDLINE search strategy

#	Search terms
1	Quality-Adjusted Life Years/
2	(quality adjusted or adjusted life year).ti,ab,kf.
3	(qaly or qald or qale or qtime).ti,ab,kf.
4	(illness state1 or health state1).ti,ab,kf.
5	(hui or hui1 or hui2 or hui3).ti,ab,kf.
6	(multiattribute or multi attribute).ti,ab,kf.
7	(utility adj3 (score1 or valu or health or cost or measur or disease or mean or gain or gains or index)).ti,ab,kf.
8	utilities.ti,ab,kf.
9	(eq-5d or eq5d or eq-5 or eq5 or euro qual or euroqual or euro qual5d or euroqual5d or euro qol or euroqol or euro qol5d or euroqol5d or euro quol or euroquol or euro quol5d or euroquol5d or eur qol or eurqol or eur qol5d or eur qol5d or eur?qul or eur?qul5d or euro quality of life or european qol).ti,ab,kf.
10	(euro adj3 (5 d or 5d or 5 dimension or 5 domain or 5domain)).ti,ab,kf.
11	(sf36 or sf 36 or sf thirtysix or sf thirty six).ti,ab,kf.
12	(time trade off1 or time tradeoff1 or tto or timetradeoff1).ti,ab,kf.
13	adolescen*.tw.
14	youth*.tw.
15	child*.tw.
16	young person*.tw.
17	teen*.tw.
18	(young adj (adult* or people* or person* or girl* or boy*)).tw.
19	(pediatric or paediatric or pediatrics or paediatrics).tw.
20	**or/**1-12
21	**and** 13

## Appendix B Included studies

**Table 5:** Included studies

First author	Title
Beusterien, K (2012) [36]	Development of the multi-attribute Adolescent Health Utility Measure (AHUM)
Chiou (2005) [7]	Development of the multi-attribute Pediatric Asthma Health Outcome Measure (PAHOM)
Hettiarachchi, RM (2023) [8]	Developing an Australian utility value set for the Early Childhood Oral Health Impact Scale-4D (ECOHIS-4D) using a discrete choice experiment
Kulkarni, A (2003) [37]	A disease-specific health status measurement for children with hydrocephalus
Kulkarni, A (2004) [38]	Measuring the health status of children with hydrocephalus by using a new outcome measure
Oluboyede, Y (2013) [9]	Quality of life assessment in adolescent obesity: Development of a new instrument for economic evaluation
Payakachat, N (2014) [39]	Predicting health utilities for children with autism spectrum disorders
Petrou, S (2014) [40]	Economic evaluation of nebulized magnesium sulphate in acute severe asthma in children
Reckers-Droog, V (2020) [41]	Presentation and validation of the Abbreviated Self Completion Teen-Addiction Severity Index (ASC T-ASI): A preference-based measure for use in health-economic evaluations
Retzler, J (2018) [21]	Utility elicitation in adults and children for allergic rhinoconjunctivitis and associated health states
Robinson, T (2019) [4]	Estimating CHU-9D Utility Scores from the WAItE: A Mapping Algorithm for Economic Evaluation
Rogers, H (2019) [10]	Adolescent valuation of CARIES-QC-U: a child-centred preference-based measure of dental caries
Schawo, S (2019) [42]	Obtaining Preference Scores for an Abbreviated Self-Completion 209 Version of the Teen-Addiction Severity Index (ASC T-ASI) to Value Therapy Outcomes of Systemic Family Interventions: a Discrete Choice Experiment
Sharma, R (2019) [43]	Mapping the Strengths and Difficulties Questionnaire onto the Child Health Utility 9D in a large study of children
Stevens, K. (2005) [44]	The development of a preference-based measure of health in children with atopic dermatitis
Tonmukayakul, U (2020) [45]	Health-related quality of life and upper-limb impairment in children with cerebral palsy: developing a mapping algorithm
Wong, C (2017) [46]	Mapping the SRS-22r questionnaire onto the EQ-5D-5L utility score in patients with adolescent idiopathic scoliosis
Wright, D (2016) [47]	The Costs and Cost-effectiveness of Collaborative Care for Adolescents With Depression in Primary Care Settings: A Randomized Clinical Trial

## Materials availability

All materials generated or analysed during this study are included in this published article (and its supplementary information files).

## Code availability

No programming code was used during the production of this manuscript.
