# Supplementary material for: A systematic review of condition-specific preference-based measures used in young people and their valuation methods
Source: J Patient Rep Outcomes. 2024 Dec 19;8:151. doi: 10.1186/s41687-024-00826-5 (PMC11659529; doi:10.1186/s41687-024-00826-5)
Supplement: Supplementary file 2 — Supplementary Material 2 [file 41687_2024_826_MOESM2_ESM.docx]

**Table S1 - The RETRIEVE long checklist**

This checklist is modular, not all sections will apply to all papers.

| **Section A - Stated preferences considered relevant to valuing child HRQoL and sample characteristics** | |
| --- | --- |
| **A1 – Stated preferences** | |
| A1a | Whose preferences were sought?   - Adults *Go to A2* - Children and young people (CYP) <18 years *Go to A3* - Mixed adults and CYP *Complete A2*   *and A3* |
| A1b | Did the authors provide a rationale for whose preference were sought?   - Yes - No |
| **A2 Adults’ stated preferences** | |
| A2a | Which adults were the focus of preference elicitation?   - General population - Parent or caregiver of child - Health care professionals - Adult with a health condition - Other adults, please specify |
| A2b | What perspective were adults asked to take in considering the child states to be valued? e.g. thinking about the health states as experienced by:   - Own child (parent) - Another child they know - A hypothetical child - Their own health, thinking back to when they were a child - Their own health, as if they were a child now - Their own health, but blinded to the states under consideration being specific to children - Person with a health condition (e.g. a health professional asked to take the person with a health condition’s perspective) - Other, please specify: |
| A2c | Was the age of the child, for whom respondents were asked to imagine health states to be valued, specified?   - Yes Go to A2d - No Go to A4 - Not applicable Go to A4 |
| A2d | If yes, what was the age of the child? |
| A2e | Was the rationale for the choice of the age of child provided?   - Yes - No |
| **A3 Children and young people’s stated preferences** | |
| A3a | From which child/young person were preferences elicited?   - General population - Person with a health condition |

|  | - Other children, please specify: |
| --- | --- |
| A3b | What perspective was the (child/young person) respondent asked to take? e.g. thinking about the health states as experienced by:   - Themselves (i.e. their own perspective) - Another known child - A hypothetical child - Other, please specify: |
| A3c | Was the age of the child/young person, for whom respondents were asked to imagine health states to be valued, specified?   - Not applicable (i.e. own perspective/themselves) Go to A4 - It was applicable but not stated Go to A3f - Yes Go to A3e |
| A3d | If the age was specified, what was the age? |
| A3e | Was the rationale for the choice of the age of child/young person provided?   - Yes - No |
| **A4 Sample** | |
| A4a | Was the population or sample frame defined from which the sample was drawn? (e.g., country, age, condition)   - Yes - No |
| A4b | Is information provided on how the sample was recruited (e.g., doorknocking, location, online panel, convenience sample)?   - Yes - Partial - No |
| A4c | If data were collected online, were efforts made to avoid on-line panel fraud?   - Yes - No - Not applicable |
| A4d | Was there a target sample size (or sample sizes if by block – e.g. number of tasks per block (e.g. DCE) or health state (e.g. TTO))?   - Yes - No Go to A4g |
| A4e | Was the target sample justified?   - Yes - No |
| A4f | Was the target sample achieved?   - Yes - No - Unclear |
| A4g | Were the characteristics of the final sample described?   - Yes - No Go to A4i |

|  |  |
| --- | --- |
| A4h | Did the sample characteristics match the intended population?   - Yes - No - Unclear |
| A4i | Was the year the data collected stated?   - Yes – what year(s) were the data collected? - No |
| A4j | Was information provided on missing data? (non-completion, withdrawals)?   - Yes - Partial - No |

| **Section B - Child HRQoL states to be valued** | |
| --- | --- |
| **B1 Type of study** | |
| B1 | Did the values reported in this paper comprise:   - A value set? *Go to B2* - Values for a limited number of health states (e.g. vignette)? *Go to B3* |
| **B2 Value Sets** | |
| B2a | Which HRQoL instrument was valued? |
| B2b | Were the domains and response options of the instrument clearly described?   - Yes - No |
| B2c | What experimental design approach was used to choose the health states (combination of  dimension levels) to be valued? |
| B2d | How were the health states assigned to respondents? |
| **B3 Specific health states** | |
| B3a | How were the health states described?   - Disease specific vignettes - From a disease-specific HRQoL instrument - Other, please specify |
| B3b | How many health states were preferences elicited for? 14 |
| B3c | Was the rationale for the selection of these health states specified?   - Yes – What was the rationale? Vignettes were designed and developed based on current clinical guidelines. - No |

| **Section C – Methods used to elicit stated preferences for child HRQoL** | |
| --- | --- |
| **C1** | **Which method or methods were used to elicit stated preferences?**   - DCE - TTO - SG - BWS |

|  | - VAS - Other, please specify | |
| --- | --- | --- |
| **C2** | **Was a rationale for the choice of method(s) provided?**   - Yes - No | |
| C2a | If yes, what was the rationale? Standard gamble was used in adults as this is the only technique that truly reflects decisions under uncertainty. Visual analogue scale was used in children because of comprehension issues with the standard gamble and the use of the comparator death. | |
| **C3** | **Was the duration of the states to be valued reported (e.g ‘x years in this state, followed by death’)?**   - Yes - No Go to C4 | |
| C3a | Was the duration fixed?   - Yes - No | |
| C3b | What duration(s) was used? | |
| **C4** | **Did the method(s) allow values to be elicited that were < 0 (‘worse than dead’)?**  □   - Yes Go to C5 - No | |
| C4a | How were values < 0 elicited? | |
| C4b | What was the minimum value possible? (may vary according to the method used so should be clearly stated) | |
| C4c | What determined how the task was terminated? | |
| **C5** | **How were the values anchored on a utility scale?** | |
| **C6** | **What was the mode of administration for the stated preference tasks?**   - Online self-completion by the respondent - Self-completion of mailed questionnaires - Online computer assisted personal interview (CAPI) - In person CAPI - In person interview - Other, please specify | |
| **C7** | **How was the quality of stated preference data assessed?** Responses thought to be lacking face validity were removed as they were indicative of participant not responding in a thoughtful manner or having misunderstood the question. | |
| **C8** | **Were any exclusions made to the preference data (eg used to represent average preferences)?**   - Yes - No - Unclear | Go to C9 Go to C9 |
| C8a | **Were reasons for the exclusions provided?** | |

|  | - Yes - No - Unclear |
| --- | --- |
| **C9** | **Were the health states randomly assigned?**   - Yes - No - Unclear |
| **C10** | **Was ethics approval for the study obtained from an appropriate research ethics committee?**   - Yes - No - Unclear - Not stated |
| **C11** | **Were sources of funding and non-monetary support and the role of the funder(s) in the design described?**   - Yes - No |

| **Section D – Econometric modelling and statistical methods** | |
| --- | --- |
| **D1 – Did the values reported comprise:** | |
|  | - A value set? Go to D2 - values for a limited number of health states (vignette or Go to D3 condition-specific)? |
| **D2 Econometric modelling of value sets for HRQoL instruments** | |
| D2a | What was the theoretical model? OR What models were estimated? NA |
| D2b | Were the main assumptions of the model stated? (e.g. assumptions about preference homogeneity/heterogeneity)   - Yes - No - Unclear |
| D2c | How was the constant term treated (if included)? NA |
| D2d | How were missing data handled (e.g.: imputation, complete case analysis) Complete case analysis |
| D2e | Were subgroup analyses completed?   - Yes - No - Not applicable |
| D2f | Were interaction terms included?   - Yes - No *If no, go to D2h* |
| D2g | Were details of the interactions provided?   - Yes - No - Not applicable |

| D2h | Were non-linear specifications considered?   - Yes - No | |
| --- | --- | --- |
| D2i | Was more than one model described?   - Yes - No | *If no, go to D2m* |
| D2j | Were goodness-of-fit statistics for each model reported?   - Yes - No | |
| D2k | Was the preferred model clearly stated?   - Yes - No | |
| D2l | Were the criteria used to select the preferred model described?   - Yes - No | |
| D2m | Do the preference parameters for the health states follow a logical order (monotonic)?   - Yes *If yes, go to D2p* - No | |
| D2n | Was any post estimation undertaken to force monotonicity (e.g. collapsing levels)?   - Yes - No - Unclear/not stated | |
| D2o | How were insignificant differences between adjacent levels managed (e.g. collapsed/ forced to be different)? Not managed | |
| D2p | Were robustness checks conducted?   - Yes - No | |
| D2q | Was uncertainty around values reported?   - Yes - No | |
| **D3 Analysis of values for specific HRQoL states** | | |
| D3a | Have the statistical methods been described?   - Yes - No | *If no, go to D3c* |
| D3b | Have the statistical methods been justified?   - Yes - No | |
| D3c | How were missing data handled (e.g.: imputation, complete case analysis)? Complete case analysis | |
| D3d | Have subgroup analyses and interactions been undertaken?   - Yes - No | *If no, go to D3h* |

| D3e | Were sub-groups and interaction variable chosen for assessment justified?   - Yes - No |
| --- | --- |
| D3f | Were sensitivity analyses undertaken?   - Yes *If no, go to Section E* - No |
| D3g | Were sensitivity analyses described?   - Yes - No |

| **Section E - Characteristics of values** | |
| --- | --- |
| **E1** | **Was there qualitative or quantitative evidence reported that demonstrates the extent to which respondents engaged with and understood the valuation tasks?**   - Yes - No |
| **E2** | **Where a value was reported, were the values generated by the final model logically consistent?**   - Yes - No - Unclear |
| **E3** | **Did authors report the distribution of values over all states defined by the HRQoL instrument (e.g. as per Figure 1 from Pan et al 2022, showen below)**   - Yes - No |
| **E4** | **Key characteristics of the values** |
| E4a | How many percentage values less than zero were possible? |
| E4b | What was the maximum possible value less than one? 0.874 |
| E4c | Where in the descriptive system does the biggest change in values occur, when shifting between adjacent states? |
| **E5** | **Was the order of importance of dimensions (domains) suggested by the value set discussed?**   - Yes - No |
