# Supplementary material for: A systematic review of condition-specific preference-based measures used in young people and their valuation methods
Source: J Patient Rep Outcomes. 2024 Dec 19;8:151. doi: 10.1186/s41687-024-00826-5 (PMC11659529; doi:10.1186/s41687-024-00826-5)
Supplement: Supplementary file 5 — Supplementary Material 5 [file 41687_2024_826_MOESM5_ESM.docx]

**Table S1 - The RETRIEVE long checklist**

This checklist is modular, not all sections will apply to all papers.

| **Section A - Stated preferences considered relevant to valuing child HRQoL and sample characteristics** | |
| --- | --- |
| **A1 – Stated preferences** | |
| A1a | Whose preferences were sought?   - Adults *Go to A2* - Children and young people (CYP) <18 years *Go to A3* - Mixed adults and CYP *Complete A2*   *and A3* |
| A1b | Did the authors provide a rationale for whose preference were sought?   - Yes - No |
| **A2 Adults’ stated preferences** | |
| A2a | Which adults were the focus of preference elicitation?   - General population - Parent or caregiver of child - Health care professionals - Adult with a health condition - Other adults, please specify |
| A2b | What perspective were adults asked to take in considering the child states to be valued? e.g. thinking about the health states as experienced by:   - Own child (parent) - Another child they know - A hypothetical child - Their own health, thinking back to when they were a child - Their own health, as if they were a child now - Their own health, but blinded to the states under consideration being specific to children - Person with a health condition (e.g. a health professional asked to take the person with a health condition’s perspective) - Other, please specify: |
| A2c | Was the age of the child, for whom respondents were asked to imagine health states to be valued, specified?   - Yes Go to A2d - No Go to A4 - Not applicable Go to A4 |
| A2d | If yes, what was the age of the child? |
| A2e | Was the rationale for the choice of the age of child provided?   - Yes - No |
| **A3 Children and young people’s stated preferences** | |
| A3a | From which child/young person were preferences elicited?   - General population - Person with a health condition |

|  | - Other children, please specify: |
| --- | --- |
| A3b | What perspective was the (child/young person) respondent asked to take? e.g. thinking about the health states as experienced by:   - Themselves (i.e. their own perspective) - Another known child - A hypothetical child - Other, please specify: |
| A3c | Was the age of the child/young person, for whom respondents were asked to imagine health states to be valued, specified?   - Not applicable (i.e. own perspective/themselves) Go to A4 - It was applicable but not stated Go to A3f - Yes Go to A3e |
| A3d | If the age was specified, what was the age? |
| A3e | Was the rationale for the choice of the age of child/young person provided?   - Yes - No |
| **A4 Sample** | |
| A4a | Was the population or sample frame defined from which the sample was drawn? (e.g., country, age, condition)   - Yes - No |
| A4b | Is information provided on how the sample was recruited (e.g., doorknocking, location, online panel, convenience sample)?   - Yes - Partial - No |
| A4c | If data were collected online, were efforts made to avoid on-line panel fraud?   - Yes - No - Not applicable |
| A4d | Was there a target sample size (or sample sizes if by block – e.g. number of tasks per block (e.g. DCE) or health state (e.g. TTO))?   - Yes - No Go to A4g |
| A4e | Was the target sample justified?   - Yes - No |
| A4f | Was the target sample achieved?   - Yes - No - Unclear |
| A4g | Were the characteristics of the final sample described?   - Yes - No Go to A4i |

|  |  |
| --- | --- |
| A4h | Did the sample characteristics match the intended population?   - Yes - No - Unclear |
| A4i | Was the year the data collected stated?   - Yes – what year(s) were the data collected? - No |
| A4j | Was information provided on missing data? (non-completion, withdrawals)?   - Yes - Partial - No |

| **Section B - Child HRQoL states to be valued** | |
| --- | --- |
| **B1 Type of study** | |
| B1 | Did the values reported in this paper comprise:   - A value set? *Go to B2* - Values for a limited number of health states (e.g. vignette)? *Go to B3* |
| **B2 Value Sets** | |
| B2a | Which HRQoL instrument was valued? CARIES-QC |
| B2b | Were the domains and response options of the instrument clearly described?   - Yes - No |
| B2c | What experimental design approach was used to choose the health states (combination of  dimension levels) to be valued?  BWS = Full factorial design  DCE = d-efficient design |
| B2d | How were the health states assigned to respondents?  NS |
| **B3 Specific health states** | |
| B3a | How were the health states described?   - Disease specific vignettes - From a disease-specific HRQoL instrument - Other, please specify |
| B3b | How many health states were preferences elicited for? 243 |
| B3c | Was the rationale for the selection of these health states specified?   - Yes – What was the rationale? The same approach that was used in the valuation of the EQ5D-Y - No |

| **Section C – Methods used to elicit stated preferences for child HRQoL** | |
| --- | --- |
| **C1** | **Which method or methods were used to elicit stated preferences?**   - DCE - TTO - SG - BWS |

|  | - VAS - Other, please specify | |
| --- | --- | --- |
| **C2** | **Was a rationale for the choice of method(s) provided?**   - Yes - No | |
| C2a | If yes, what was the rationale? Emerging evidence suggests that adolescents have a greater understanding of BWS tasks compared to DCE | |
| **C3** | **Was the duration of the states to be valued reported (e.g ‘x years in this state, followed by death’)?**   - Yes - No Go to C4 | |
| C3a | Was the duration fixed?   - Yes - No | |
| C3b | What duration(s) was used? 6 months, 1 year, 1 year 6 months, 2 years | |
| **C4** | **Did the method(s) allow values to be elicited that were < 0 (‘worse than dead’)?**  □   - Yes Go to C5 - No | |
| C4a | How were values < 0 elicited? | |
| C4b | What was the minimum value possible? (may vary according to the method used so should be clearly stated) 0 | |
| C4c | What determined how the task was terminated? NS | |
| **C5** | **How were the values anchored on a utility scale?** DCETTO | |
| **C6** | **What was the mode of administration for the stated preference tasks?**   - Online self-completion by the respondent - Self-completion of mailed questionnaires - Online computer assisted personal interview (CAPI) - In person CAPI - In person interview - Other, please specify | |
| **C7** | **How was the quality of stated preference data assessed?** Dominance test and excluding participants who were too slow or too fast to complete the task. | |
| **C8** | **Were any exclusions made to the preference data (eg used to represent average preferences)?**   - Yes - No - Unclear | Go to C9 Go to C9 |
| C8a | **Were reasons for the exclusions provided?** | |

|  | - Yes - No - Unclear |
| --- | --- |
| **C9** | **Were the health states randomly assigned?**   - Yes - No - Unclear |
| **C10** | **Was ethics approval for the study obtained from an appropriate research ethics committee?**   - Yes - No - Unclear - Not stated |
| **C11** | **Were sources of funding and non-monetary support and the role of the funder(s) in the design described?**   - Yes - No |

| **Section D – Econometric modelling and statistical methods** | |
| --- | --- |
| **D1 – Did the values reported comprise:** | |
|  | - A value set? Go to D2 - values for a limited number of health states (vignette or Go to D3 condition-specific)? |
| **D2 Econometric modelling of value sets for HRQoL instruments** | |
| D2a | What was the theoretical model? OR What models were estimated? Conditional logistic regression |
| D2b | Were the main assumptions of the model stated? (e.g. assumptions about preference homogeneity/heterogeneity)   - Yes - No - Unclear |
| D2c | How was the constant term treated (if included)? Not included in the BWS model |
| D2d | How were missing data handled (e.g.: imputation, complete case analysis) NS |
| D2e | Were subgroup analyses completed?   - Yes - No - Not applicable |
| D2f | Were interaction terms included?   - Yes - No *If no, go to D2h* |
| D2g | Were details of the interactions provided?   - Yes - No - Not applicable |

| D2h | Were non-linear specifications considered?   - Yes - No | |
| --- | --- | --- |
| D2i | Was more than one model described?   - Yes - No | *If no, go to D2m* |
| D2j | Were goodness-of-fit statistics for each model reported?   - Yes - No | |
| D2k | Was the preferred model clearly stated?   - Yes - No | |
| D2l | Were the criteria used to select the preferred model described?   - Yes - No | |
| D2m | Do the preference parameters for the health states follow a logical order (monotonic)?   - Yes *If yes, go to D2p* - No | |
| D2n | Was any post estimation undertaken to force monotonicity (e.g. collapsing levels)?   - Yes - No - Unclear/not stated | |
| D2o | How were insignificant differences between adjacent levels managed (e.g. collapsed/ forced to be different)?  Any inconsistent adjacent levels were merged to produce a single utility decrement that can be applied when the dimension is at either of the respective levels | |
| D2p | Were robustness checks conducted?   - Yes - No | |
| D2q | Was uncertainty around values reported?   - Yes - No | |
| **D3 Analysis of values for specific HRQoL states** | | |
| D3a | Have the statistical methods been described?   - Yes - No | *If no, go to D3c* |
| D3b | Have the statistical methods been justified?   - Yes - No | |
| D3c | How were missing data handled (e.g.: imputation, complete case analysis)? | |
| D3d | Have subgroup analyses and interactions been undertaken?   - Yes - No | *If no, go to D3h* |

| D3e | Were sub-groups and interaction variable chosen for assessment justified?   - Yes - No |
| --- | --- |
| D3f | Were sensitivity analyses undertaken?   - Yes *If no, go to Section E* - No |
| D3g | Were sensitivity analyses described?   - Yes - No |

| **Section E - Characteristics of values** | |
| --- | --- |
| **E1** | **Was there qualitative or quantitative evidence reported that demonstrates the extent to which respondents engaged with and understood the valuation tasks?**   - Yes - No |
| **E2** | **Where a value was reported, were the values generated by the final model logically consistent?**   - Yes - No - Unclear |
| **E3** | **Did authors report the distribution of values over all states defined by the HRQoL instrument (e.g. as per Figure 1 from Pan et al 2022, showen below)**   - Yes - No |
| **E4** | **Key characteristics of the values** |
| E4a | How many percentage values less than zero were possible? 0 |
| E4b | What was the maximum possible value less than one? 0.942 |
| E4c | Where in the descriptive system does the biggest change in values occur, when shifting between adjacent states? Hurt1 to Hurt2 |
| **E5** | **Was the order of importance of dimensions (domains) suggested by the value set discussed?**   - Yes - No |
